# Investigation of the Accuracy and Contributing Factors of AI‐Based Diagnosis of Urothelial Carcinoma in Canine Abdominal Radiography

**DOI:** 10.1111/vru.70172

**Published:** 2026-04-20

**Authors:** Masahiro Murakami, Kosuke Kinoshita, Mario Sola, Anthony Lutz

**Affiliations:** ^1^ Department of Veterinary Clinical Sciences College of Veterinary Medicine Purdue University West Lafayette Indiana USA; ^2^ Department of Comparative Pathobiology College of Veterinary Medicine Purdue University West Lafayette Indiana USA; ^3^ Vetology Innovations, LLC San Diego California USA

**Keywords:** artificial intelligence, explainable AI, transitional cell carcinoma, x‐ray

## Abstract

Urothelial carcinoma (UC) is a highly malignant urinary cancer of the transitional epithelium in dogs. Recent advances in artificial intelligence (AI) and machine learning have shown substantial potential in veterinary medicine. The purpose of this study was to evaluate the accuracy of AI‐based software in detecting UC in dogs using abdominal radiography and to identify factors that influence the sensitivity of AI‐based diagnosis. Dogs underwent abdominal radiography, and ultrasound was retrospectively retrieved. Dogs with histologically confirmed UC and ultrasound changes were included in UC training and UC validation groups, whereas dogs without clinical suspicion of urinary neoplasia and without ultrasound findings consistent with UC were included as non‐UC training and non‐UC validation groups. Histological and imaging findings of UC were recorded. A convolutional neural network (CNN) was trained with 500 studies from the UC training and 500 studies from the non‐UC training groups. For validation, an additional 185 studies from the UC validation and 180 studies from the non‐UC validation groups were used to provide AI‐based diagnosis of UC by the trained CNN. The sensitivity, specificity, and accuracy of the AI‐based diagnosis of UC were 69%, 67%, and 68%, respectively. The software showed higher sensitivity in detecting more severe UC with mineralization. However, medial iliac lymphadenomegaly and ureteral obstruction did not improve the sensitivity of AI‐based diagnosis. In conclusion, well‐trained CNN demonstrated moderate accuracy for detecting UC using abdominal radiographs, with higher sensitivity in cases with more advanced disease. The unexpectedly superior performance of the ventrodorsal (VD) view warrants further investigation.

**Abbreviations**
AI: artificial intelligence
CNN: convolutional neural network
ML: machine learning
TCC: transitional cell carcinoma
UC: urothelial carcinoma
VD: ventrodorsal

## 1 Introduction

Urothelial carcinoma (UC), also known as transitional cell carcinoma (TCC), is a highly malignant neoplasm that primarily affects the transitional epithelium of the urinary tract. The incidence of UC in dogs is estimated to be 1%–2% in the general dog population [1, 2, 3]. The etiology of UC is multifactorial, with contributions from both genetic factors and environmental exposures [3]. Identified risk factors include sex, with females at higher risk, neutered status, obesity, specific breeds, and exposure to certain pesticides and herbicides [2, 4, 5]. In particular, Scottish Terriers have an 18‐fold increased risk of UC compared to mixed‐breed dogs [3]. Similarly, West Highland White Terriers and Shetland Sheepdogs have been reported to have an increased risk [3]. A retrospective analysis found a female‐to‐male ratio of 1.7:1 among dogs with UC [3], suggesting a sex predisposition.

The clinical prognosis for dogs with UC remains poor, as evidenced by a median survival of 251 days post‐diagnosis [6]. Distant metastasis is a common and challenging complication; in fact, more than 50% of dogs with UC are found to have distant metastasis at the time of death, severely compromising their prognosis [2]. Diagnostic approaches for UC are varied and commonly include diagnostic imaging in addition to histopathologic evaluation. Tumor samples can be obtained by cystoscopy, traumatic catheterization, or laparotomy with subsequent cystotomy [3, 6, 7]. Prognostic assessment is based on imaging features, with ultrasound evidence of urinary bladder wall muscular invasion, heterogeneity within the mass, and involvement of the trigone region of the urinary bladder being associated with decreased survival [8]. In addition, radiographic findings of lumbar metastatic spondylitis, prostatomegaly, or enlarged regional lymph nodes are reported in dogs with UC.

Recent advances in artificial intelligence (AI) and machine learning (ML) technologies have shown remarkable potential for improving diagnostic efficiency in both medical and veterinary medicine [9, 10, 11]. These technologies, particularly in the area of image analysis, enable the differentiation of specific features across a range of medical conditions and imaging techniques [12, 13, 14, 15, 16]. In human medicine, AI applications support the initial interpretation of medical radiographs, with algorithms designed to identify thoracic conditions, such as pneumothorax and malignant neoplasms, and extrathoracic abnormalities, including bone fractures [17, 18, 19]. In addition, AI has enhanced mammography analysis to facilitate early detection of breast cancer, potentially reducing the workload of radiologists [20]. Similarly, in veterinary medicine, AI software has been reported to detect pleural effusions and hip dysplasia with high accuracy [21, 22]. Despite these advances, research into the application of deep learning to veterinary diagnostic imaging is relatively limited, suggesting an area for further investigation.

The purpose of this study was (1) to quantify the diagnostic performance of a convolutional neural network (CNN) for identifying canine UC/TCC using routine abdominal radiographs, using histopathology as the reference standard, and (2) to explore, a priori, whether model sensitivity varies with clinically relevant indicators of disease extent and severity, thereby providing an interpretable context for model strengths and failure modes. We hypothesized that overall performance would be moderate because radiographic signs of UC can be subtle or absent, but that sensitivity would be higher in cases with radiographically apparent mineralization and other markers of advanced disease. We also evaluated projection‐specific performance (lateral vs. ventrodorsal [VD]) to assess whether view‐dependent information influences model predictions.

## 2 Materials and Methods

### 2.1 Study Design and Case Population

We conducted a retrospective analysis of diagnostic accuracy using the Purdue University Veterinary Hospital Medical Record database from January 1, 2017, to January 2023. The study population included canine patients who underwent abdominal radiography and abdominal ultrasonography within 24 h of one another.

### 2.2 Ethical Considerations

Given the retrospective nature of the study, using data from routine clinical practice, formal ethics committee approval was not required. However, informed consent for the processing of personal data was obtained from all participating dog owners.

### 2.3 Data Selection and Recording of Patient Information

Under the guidance of an American College of Veterinary Radiology (ACVR) board‐certified radiologist (M.M.), a veterinarian (K.K.) performed case selection and categorized cases into four groups: UC training, UC validation, non‐UC training, and non‐UC validation. Inclusion criteria for UC groups required a minimum of two orthogonal abdominal radiographs per study, and histopathologic confirmation of UC or TCC. In this study, abdominal ultrasound was performed in all cases as part of routine clinical evaluation, and ultrasound‐derived variables were used in secondary analyses. The UC group was divided into a training group (UC training, consisting of 500 studies) and a validation group (UC validation). The non‐UC groups included dogs without lower urinary tract neoplasia, either clinically or ultrasonographically, matched by age to the UC groups. Cases with cystolithiasis or other diseases were not excluded unless there was a suspicion of lower urinary tract neoplasia. Recruitment continued until the non‐UC groups were comparable in size to the UC groups.

A board‐certified radiologist (M.M.) and a veterinarian (K.K.) reviewed the ultrasound images and corresponding abdominal radiographs, as well as the medical records, including histology report. Data recorded included patient age, along with detailed information on UC: histological grade, organ involved, specific location within the urinary bladder (if present in the urinary bladder), presence of mineralization, medial iliac lymphadenomegaly (sublumbar region), ureteral obstruction at the ureterovesical junction, muscular invasion, and lumbar metastatic spondylitis.

### 2.4 Histopathology and Grading

Histologic diagnosis of UC/TCC was established on routine H&E preparations by board‐certified pathologists. Grading primarily followed a canine two‐tier scheme (low vs. high grade) based on cellular morphology and growth patterns [23]. For institutional comparability, cases were also mapped to a four‐tier system adapted from human pathology [24]; this four‐tier mapping has not been validated for direct applicability in dogs. In this mapping, Grades 1 and 2 correspond to low‐grade and Grades 3 and 4 to high‐grade carcinoma. Operational features included Grade 1—papillary or flat; ≥7 cell layers; orderly maturation; minimal atypia; rare mitoses; no invasion, Grade 2—mild cellular atypia and nuclear pleomorphism with visible nucleoli, Grade 3—loss of polarity; moderate atypia/pleomorphism; clumped chromatin; visible nucleoli; invasion into lamina propria may be present, and Grade 4—disorganized architecture; marked atypia/pleomorphism; frequent (including atypical) mitoses; invasion beyond lamina propria into muscularis. Because small cystoscopic biopsies can underestimate invasion, muscular invasion was additionally abstracted from ultrasound or surgical reports when reported.

### 2.5 Data Collection and Processing for AI Training

DICOM files of abdominal radiographs were collected from two different groups: the UC training group and the non‐UC training group. Once collected, the DICOM files were securely transmitted to the AI program engineer (A.L.) for training of the Vetology AI software. The transferred datasets included identifiers indicating the group affiliation of each image (UC or non‐UC) but intentionally excluded any patient history, identifying information, and accompanying radiology reports. Each radiograph was labeled with the radiographic view it represented.

In cases where multiple radiographs were available for the same radiographic view, a composite approach was used. All images representing the same view were used in the training dataset. When multiple radiographs existed for the same view, per‐image predicted probabilities were averaged (arithmetic mean) to obtain the view‐level prediction. In the external validation cohort (*n* = 365 studies), lateral projections comprised 466 images; 254/365 studies (69.6%) had a single lateral image; and 111/365 (30.4%) had ≥2 lateral images (e.g., multiple exposures required to include the entire abdomen and/or bilateral lateral recumbency projections). VD projections comprised 447 images; 241/365 studies (66.0%) had a single VD image; and 124/365 (34.0%) had ≥2 VD images (e.g., multiple exposures required to include the entire abdomen).

Preprocessing was fully automated using Vetology's in‐house library. Steps included intensity normalization (OpenCV normalize, scaling pixel values to 0–255), denoising via OpenCV fastNlMeansDenoising, and gamma correction that brightened pixels in the lowest quartile and darkened those in the highest quartile to mitigate under‐/overexposure. No manual per‐image adjustments were performed.

### 2.6 AI Training

The software used in this study classified images into two groups: UC and non‐UC. The CNN was trained on 500 abdominal radiograph studies from dogs with UC (UC training group) and 500 abdominal radiograph studies from dogs without UC (non‐UC training group) using the TensorFlow 2.6 Python library. TensorFlow is an open‐source platform used for ML that provides a user interface for executing AI algorithms. For the training of Vetology AI software, initial weights were produced by pretraining the model on 20,000 unlabeled canine images with the SimCLR framework. This contrastive learning method optimizes the initial training weights to detect relevant features in the radiograph and boosts performance when training with smaller datasets. We used a modified EfficientNetV2 (B0) network architecture, importing the base model from the Keras library and truncating the final dense layers to allow for binary classification. The modified EfficientNetV2 (B0) model was transfer‐learned using these pretrained weights and the UC dataset.

**FIGURE 1:**
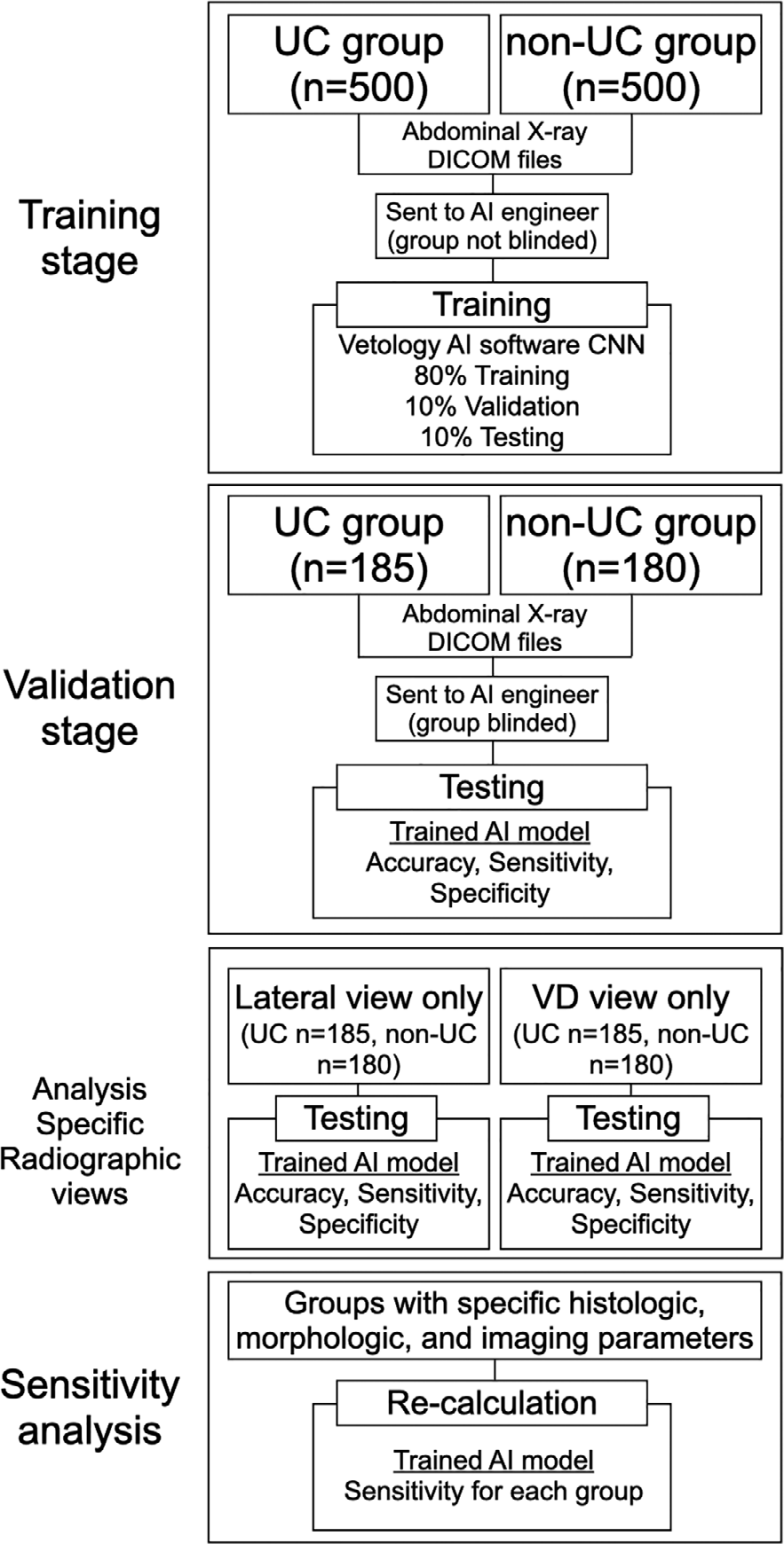
Workflow of the AI‐based diagnostic study for UC in canine abdominal radiographs. The study was divided into four stages: training stage, validation stage, analysis of specific radiographic views, and sensitivity analysis. In the training stage, abdominal radiographic DICOM files from dogs with UC (*n* = 500) and without UC (*n* = 500) were used to train the AI model using the Vetology AI software with an 80:20 split for training and validation. The validation stage included blinded testing of the trained model on new cases (UC group: *n* = 185; non‐UC group: *n* = 180) to evaluate its diagnostic performance (accuracy, sensitivity, and specificity). Specific radiographic view analysis separately tested the diagnostic ability of the model for lateral and ventrodorsal (VD) views only. Finally, sensitivity analysis was performed by recalculating diagnostic sensitivity based on histologic, morphologic, and imaging parameters. AI, artificial intelligence; CNN, convolutional neural network; UC, urothelial carcinoma.

We trained the model using an 80:10:10 split for training, validation, and testing. The validation set was a random subset (10% of the total training data), which was used for inter‐model tuning during each epoch of training. The test set was an additional random 10% subset that was then used to verify model performance one training was complete. The optimal model was chosen on the basis of the best performance (AUC, area under the curve) with respect to the holdout testing set. The final metrics for this model were then confirmed by analysis on the blind test set cases. The workflow of the AI training, validation, specific radiographic view analysis, and sensitivity analysis is depicted in Figure 1.

### 2.7 Validation

Three hundred eighty‐six abdominal radiographic studies not used during training were collected for validation (UC validation and non‐UC validation groups). The validation group consisted of 185 studies from the UC validation group and 180 studies from the non‐UC validation group. The DICOM files of the abdominal radiograph studies from these groups were transferred to the AI program engineer (A.L.) to test using trained Vetology AI software without any other information. The diagnosis of UC, as well as all patient information, was blinded to the AI program engineer (A.L.).

### 2.8 Statistical Analysis

The diagnostic ability of the trained AI program for UC was evaluated for sensitivity (TP/[TP + FN]), specificity (TN/[TN + FP]), and accuracy ((TP + TN)/[TP + TN + FP + FN]). In addition, *F*1 score and Matthews correlation coefficient (MCC) were calculated from the 2 × 2 contingency table as complementary performance metrics commonly reported for vision‐based AI. These values were derived by comparing the AI‐based diagnoses from the validation groups with the gold standard of histologic diagnosis. To determine the impact of radiographic views on diagnostic accuracy, the additional analysis was performed separately for AI‐based radiographic diagnosis using only the lateral view and those using only the VD view. To further determine the impact of UC characteristics on radiographic diagnosis, the sensitivity of AI‐based diagnosis of UC was recalculated for a variety of histologic, morphologic, and imaging parameters. These parameters included histological grade, the organ involved, specific location within the urinary bladder (if present in the urinary bladder), the presence of mineralization, medial iliac lymph node enlargement, ureteral obstruction at the ureterovesical junction, muscular invasion, and lumbar metastatic spondylitis. Ureteral obstruction status was recorded from the abdominal ultrasound report by ACVR board‐certified radiologists.

## 3 Results

A total of 1386 abdominal radiographic studies were retrospectively investigated; 685 were from dogs diagnosed with UC, and 680 were from dogs without UC. These studies were further divided into training groups (500 with UC and 500 without UC) and validation groups (185 with UC and 180 without UC). The mean age of the UC groups was 10.7 ± 2.2 years for the training group and 10.4 ± 1.9 years for the validation group.

After training on 1000 abdominal radiographic studies (500 in the UC training group and 500 in the non‐UC training group), its diagnostic performance on the validation groups showed a sensitivity of 69.0%, a specificity of 67.4%, and an accuracy of 68.2% for the detection of UC (Table 1). The corresponding *F*1 score was 0.68, and the MCC was 0.36. Analysis of the effect of specific radiographic views on the diagnostic accuracy of the trained AI program showed a sensitivity of 68.9%, a specificity of 75.4%, and an accuracy of 72.1% for the diagnosis of UC using the lateral view only. For the VD view, the sensitivity was 95.6%, the specificity was 85.1%, and the accuracy was 90.7%.

**TABLE 1:** Contingency table summarizing the diagnostic performance of the trained artificial intelligence (AI) model in the validation dataset for detecting urothelial carcinoma (UC).

Validation dataset		Trained AI model diagnosis		
		UC	Non‐UC	
Histological diagnosis	UC group	127	58	185
	Non‐UC group	59	121	180
		186	179	

*Note*: Results are presented by comparing the AI model's diagnoses (UC or non‐UC) with histological diagnoses. Sensitivity (69.0%), specificity (67.4%), accuracy (68.2%), *F*1 score (0.68), and MCC (0.36) were calculated to evaluate the model's performance.

Within the UC validation group of 185 cases, histologic grades were available for 103 cases, with 8 cases of Grade 2, 22 of Grade 3, and 74 of Grade 4 UC. The distribution of UC was in the urinary bladder in 178 studies, followed by the urethra in 105 studies and the prostate in 55 studies, with some cases showing multiorgan involvement. Of the UC cases involving the urinary bladder, the localization was as follows: 63 in the trigone/urinary bladder neck, 11 apical, 2 in the body, and 103 were diffuse.

In all 185 studies with confirmed diagnosis of UC, mineralization was present in 136, medial iliac lymph node enlargement in 36, ureteral obstruction at the ureterovesical junction in 52, muscular invasion in 87, and lumbar metastatic spondylitis in 2 studies.

**FIGURE 2:**
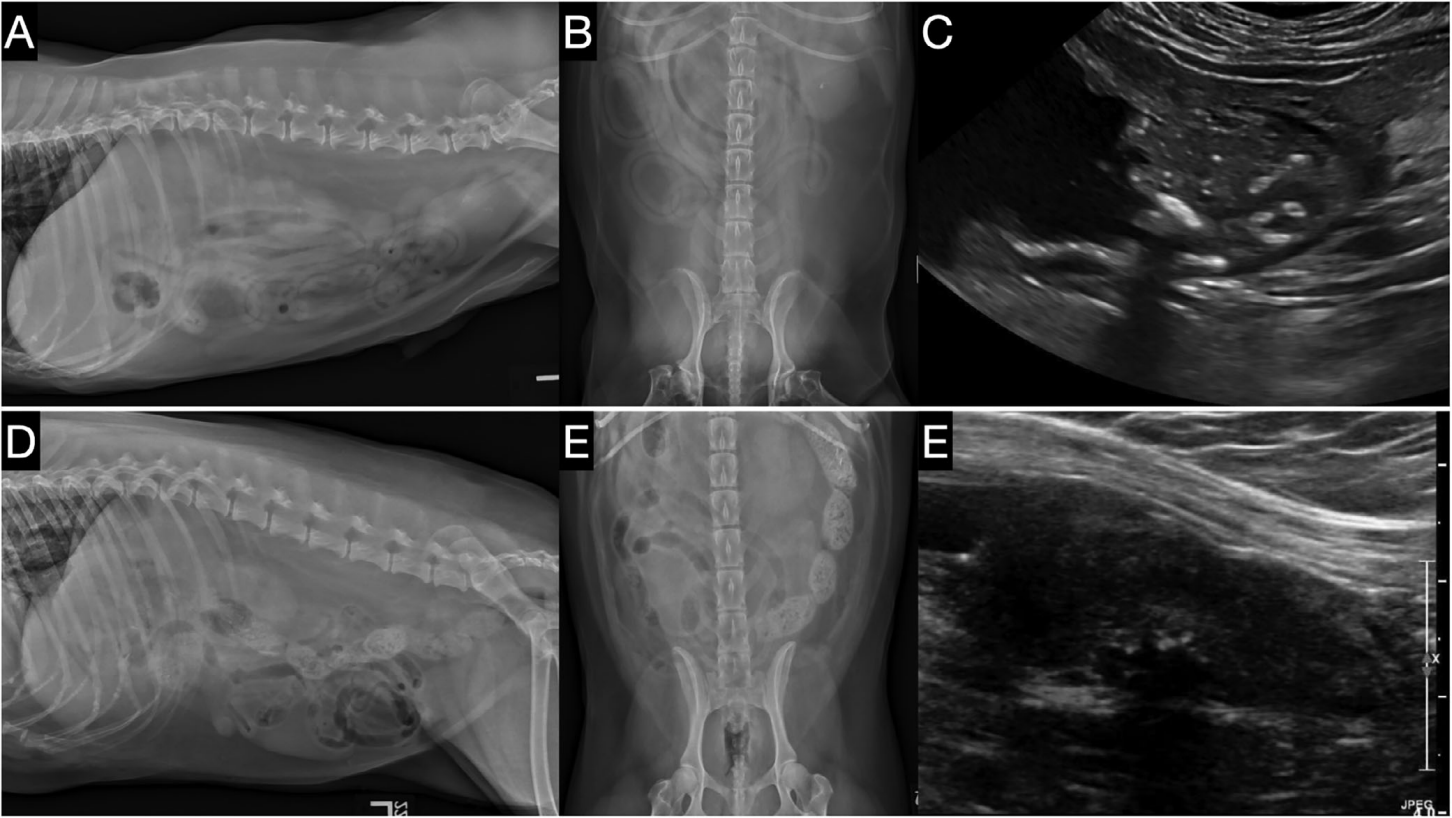
Representative radiograph and ultrasound images of canine UC cases illustrating true‐positive and false‐negative AI classifications. (A–C) (True‐positive case): (A) Left lateral abdominal radiograph shows several pinpoint mineralizations centered on the urinary bladder; no medial iliac (sublumbar region) lymphadenomegaly is present. (B) Ventrodorsal (VD) view. (C) Abdominal ultrasound confirms mineralization within a urinary bladder trigone mass consistent with urothelial carcinoma (UC); no medial iliac lymphadenomegaly is detected sonographically. The trained CNN classified this study as UC (positive). (D–F) (False‐negative case). (D) Left lateral abdominal radiograph demonstrates thickening of the urinary bladder neck extending into the proximal urethra with punctate mineralization; increased soft‐tissue opacity is present ventral to L7. (E) Ventrodorsal (VD) view. (F) Abdominal ultrasound confirms a urinary bladder‐neck/proximal urethral mass with mineralization and medial iliac lymphadenomegaly. Despite these findings and histologic confirmation of UC, the trained CNN classified this study as non‐UC (negative).

The diagnostic sensitivity of the trained AI software for UC varies according to different UC characteristics and categories, as shown in Table 2. Representative cases are shown in Figure 2. A true‐positive case (Figure 2A–C) exhibited urinary bladder mineralization without medial iliac lymphadenomegaly and was correctly classified as UC. A false‐negative case (Figure 2D–F) showed urinary bladder‐neck/proximal urethral thickening with mineralization and ultrasound‐confirmed medial iliac lymphadenomegaly, yet the model predicted non‐UC. The sensitivity for histologic grading was 63.0% for Grade 2 in eight cases, 68.2% for Grade 3 in 22 cases, and an increased sensitivity of 82.4% for Grade 4 in 74 cases. When assessing the location of UC, the trained AI software demonstrated a sensitivity of 71.3% for urinary bladder UC in 178 cases, 68.6% for urethral UC in 105 cases, and 67.3% for prostatic UC in 55 cases. Within the various urinary bladder locations, the sensitivities were 59.0% for UC involving the trigone or urinary bladder neck in 63 cases, 72.7% for apical urinary bladder UC in 11 cases, a 100% for urinary bladder body UC in two cases, and 76.7% for diffuse urinary bladder UC in 103 cases. Diagnostic sensitivity was influenced by the presence of mineralization, with a higher sensitivity of 75.7% in the presence of mineralization versus 55.1% in the absence. The sensitivity of the trained AI software was 66.7% in the presence of medial iliac lymph node enlargement in 36 cases, which increased slightly to 71.1% in the absence of enlargement in 149 cases. In cases with ureteral obstruction at the ureterovesical junction, the sensitivity was 63.5% in 52 cases, increasing to 72.4% in the absence of obstruction in 134 cases. Muscular invasion showed an increase of sensitivity, with 83.9% in 87 cases with invasion and a lower sensitivity of 58.2% in 98 cases without invasion. In the few cases with lumbar metastatic spondylitis, the trained AI software demonstrated a sensitivity of 100% in two cases, compared to a sensitivity of 70% in 183 cases without metastatic spondylitis.

**TABLE 2:** Sensitivity of the trained artificial intelligence (AI) software to diagnose urothelial carcinoma (UC) across various histologic, morphologic, and imaging parameters in the validation dataset.

Sensitivity for entire dataset: 69.0%	Specific histologic, morphologic, and imaging parameters	UC diagnosis sensitivity by trained AI model (%)
Histologic grade	Grade 2/4 (*n* = 8)	63.0
	Grade 3/4 (*n* = 22)	68.2
	Grade 4/4 (*n* = 74)	82.4
Affected organ	Urinary bladder (*n* = 178)	71.3
	Urethra (*n* = 105)	68.6
	Prostate (*n* = 55)	67.3
Location in urinary bladder	Trigone/Urinary bladder neck (*n* = 61)	59.0
	Apical urinary bladder (*n* = 11)	72.7
	Diffuse urinary bladder (*n* = 103)	76.7
Imaging findings	Mineralization + (*n* = 136)	75.7
	Mineralization − (*n* = 49)	55.1
	Muscular invasion + (*n* = 87)	83.9
	Muscular invasion − (*n* = 98)	58.2
	Medial iliac lymphadenomegaly + (*n *= 36)	66.7
	Medial iliac lymphadenomegaly − (*n *= 149)	71.1
	Urethral obstruction + (*n *= 52)	63.5
	Urethral obstruction − (*n* = 134)	72.4
	Lumbar spondylitis + (*n* = 2)	100
	Lumbar spondylitis − (*n* = 183)	70.0

*Note*: Sensitivities were calculated for histologic grading, affected organs, bladder locations within the bladder, and specific imaging findings.

## 4 Discussion

In this study, the AI program, trained on a substantial dataset of 1000 canine abdominal radiograph studies, achieved a diagnostic accuracy rate of 68.2%. Consistent with our a priori expectation that radiographic signs of UC can be subtle or absent, overall performance was moderate. Importantly, we observed increased diagnostic sensitivity of the AI‐based diagnosis when applied to cases with characteristic UC features, particularly those with histologic Grade 4/4, diffuse, muscular invasion, and mineralization. This increase in sensitivity supports the clinical value of the feature‐stratified sensitivity analyses as an interpretable way to contextualize model strengths and failure modes. A notable finding was observed when radiographic views were separated: The use of either lateral or VD views independently led to increased accuracy, with the VD view alone surpassing the lateral view in diagnostic yield. This is contrary to our initial expectations and suggests an area for further investigation.

When applying ML to medical imaging, the adequacy of the dataset size is a critical factor for the successful training of CNNs. The inherent complexity and wide range of medical images require large and diverse datasets. The dataset size has been empirically shown to have a strong impact on model performance; in general, larger datasets correlate with increased accuracy and improved diagnostic performance [15, 25]. However, the exact number of images required for model training is not a one‐size‐fits‐all approach; it depends on the complexity of the diagnostic process, the heterogeneity of the medical conditions being examined, and the types and reliability of the ground truth. Certain studies have shown a steep decline in model accuracy when the dataset per class falls below 100 images [25]. Conversely, datasets of more than 1000 images per class appear to be optimal for achieving reliable accuracy [25]. On the basis of previous publications, CNNs have been used in medical imaging, typically with training sets of more than 500 images for each class [26, 27], as for example in mammography image evaluation [28]. In veterinary medicine, where dataset availability is often limited, a recent trend has been to use datasets with more than 1000 cases per class for CNN training [21, 22, 29, 30, 31, 32]. However, it is important to note that most of these studies use ground truth from the same object that is the radiographic evaluation. In the present study, we used a dataset with approximately 500 cases in each group, diseased and non‐diseased, to train the CNN. Histopathological diagnoses were used as the definitive ground truth. The number of cases is considered sufficient for our study, especially as we are focusing on diseases that cannot be diagnosed by image analysis alone, such as radiographs.

Histopathological analysis remains the definitive diagnosis of UC in dogs [2, 3, 33]. Complementarily, ultrasonography is the preferred modality for clinical evaluation due to its ability to detect important features such as muscular invasion, vascularization, mineralization, and ureteral obstruction at the ureterovesical junction [8, 34, 35]. The ability to detect these features is critical as they may carry prognostic value. Conversely, radiography, while able to identify certain pathologic changes such as mineralization, metastatic lumbar spondylitis, medial iliac lymphadenopathy, and renomegaly due to ureteral obstruction, is generally not able to provide a clear demarcation of urinary tract tumors [2, 36]. Accordingly, we expected overall CNN performance on radiographs to be moderate rather than high. The results of our study support this hypothesis, demonstrating a sensitivity of 69.0%, a specificity of 67.4%, and an overall accuracy of 68.2%. These results support the idea that while advanced ML methods such as CNNs are promising, radiographs alone are not a reliable single diagnostic tool for UC in canine patients.

The results of the current study revealed the counterintuitive finding that CNN demonstrated greater sensitivity in the diagnosis of UC when analyzing VD views alone, compared to lateral views alone or both orthogonal views. This finding challenges the traditional belief in the primacy of lateral views for evaluation of the canine urinary bladder. Generally, the VD view is not considered optimal for evaluation of the lower urinary tract and sublumbar region due to the superimposed position of the spine, which may obscure pathologic changes in the urinary bladder, urethra, and sublumbar structures, including metastatic spondylitis and lymphadenomegaly. A possible explanation for the superior sensitivity of the VD view could be the diagnostic influence of renomegaly resulting from ureteral obstruction. However, this assumption does not hold when considering that only 52 of 185 dogs had evidence of ureteral obstruction, and furthermore, there was no increased sensitivity in the detection of UC in these particular cases compared to those without obstruction. Although we did not perform pixel‐level explainability analyses (e.g., saliency mapping) in the present study, we visually confirmed that the pixel data contained no burned‐in annotations other than standard laterality markers (L/R), which were present in both projections. Therefore, the reasons for the VD–lateral discrepancy remain uncertain and warrant prospective validation in multi‐institutional datasets, ideally incorporating explainability methods (e.g., saliency mapping and occlusion sensitivity) to localize image regions driving predictions.

Current research on CNNs in veterinary diagnostic imaging is primarily focused on refining model accuracy to augment or even surpass human diagnostic performance [10, 21, 22, 29, 30, 32]. Recognizing the limitations of radiography as a diagnostic tool for canine UC and the moderate performance of trained CNNs, our study adopts an alternative analytical strategy called feature‐stratified sensitivity analysis, which provides a clinically grounded and interpretable assessment of when the model is more likely to detect UC. By focusing on the diagnostic sensitivity associated with specific features of UC, our approach indirectly assesses the influence of these individual features on diagnostic performance, despite the overall moderate accuracy of CNNs. Our results indicate that the diagnostic sensitivity of the CNN improves when identifying UC in more advanced stages, as indicated by features such as higher histologic grades, diffuse involvement of urinary bladder, muscular invasion, and mineralization. Notably, radiographic features considered to improve accuracy, such as medial iliac lymphadenopathy and ureteral obstruction, did not affect the performance of the CNN. The potential effect of lumbar spondylitis on the diagnostic accuracy of CNN remains unclear due to its rare occurrence in our study cohort. Further research is needed to elucidate the reasons for these findings and to determine which imaging features contribute most to accurate radiographic diagnosis of UC. Dedicated follow‐up studies incorporating pixel‐level explainability methods (e.g., saliency mapping and occlusion sensitivity) and prospective multi‐reader evaluation may help clarify the image features the model uses and how these relate to clinically recognizable radiographic signs.

Our study has several limitations. First, cystolithiasis was not an exclusion criterion; therefore, mineral opacities from uroliths may have contributed to false‐positive predictions and could confound associations involving mineralization. We did not stratify performance by cystolithiasis status due to the retrospective design and the possibility of occult or small uroliths being missed on radiography and ultrasonography. Second, sex and neuter status, while available, were not analyzed because the study focused on model performance. Third, the occurrence of certain radiographic changes was limited to a small number of cases, which raises concerns about the evaluation of these features. Fourth, assessment of ureteral obstruction relied on ultrasound report text rather than standardized image re‐review due to the retrospective nature of the study. Lastly, from a modeling perspective, we selected EfficientNetV2‐B0 for its parameter efficiency and because prior internal experience suggested favorable AUC on small veterinary radiography datasets compared with older backbones. We did not perform cross‐backbone benchmarking in this cohort, which represents a methodological limitation. Finally, we did not perform pixel‐level explainability analyses to investigate why VD views outperformed lateral views; thus, the reasons for this projection‐specific discrepancy remain to be clarified. A direct comparison with radiologist performance was outside the scope of this retrospective diagnostic accuracy study and warrants a prospective, blinded multi‐reader comparison in future work. To enhance interpretability in subsequent work, we plan to implement saliency mapping (Grad‐CAM and Score‐CAM) to visualize image regions most influential to model decisions. Future work should also evaluate renal size metrics, such as kidney‐to‐L2 ratio on radiographs and subjective renomegaly, as potential predictors or modifiers of model performance in UC/TCC.

In conclusion, this study illustrates the capabilities and current limitations of AI in the diagnosis of UC in dogs using abdominal radiographs, with trained CNN demonstrating overall moderate accuracy and improved sensitivity in cases with features consistent with more advanced disease. The unexpectedly superior performance of the VD view over the lateral view warrants further research. Future studies should prospectively validate these findings in multi‐institutional datasets, investigate the reproducibility of the VD–lateral performance discrepancy, and incorporate explainability methods (e.g., saliency mapping and occlusion sensitivity) to localize image regions driving predictions. Evaluating multi‐view fusion strategies and conducting prospective blinded multi‐reader comparisons will be important to fully elucidate the role of AI in advancing veterinary diagnostic imaging.

## Author Contributions

**Anthony Lutz**: conceptualization, methodology, software, investigation, validation, formal analysis, visualization, writing – review and editing. **Masahiro Murakami**: conceptualization, methodology, data curation, investigation, validation, formal analysis, supervision, visualization, project administration, writing – original draft, writing – review and editing, resources. **Mario Sola**: methodology, investigation, validation, writing – review and editing. **Kosuke Kinoshita**: methodology, data curation, investigation, writing – original draft, writing – review and editing.

## Disclosure

Presented as an oral presentation at the 2023 Annual Scientific Conference of the American College of Veterinary Radiology. An EQUATOR network checklist was not used.

## Conflicts of Interest

The authors declare no conflicts of interest.

## Data Availability

The data that support the findings of this study are available from the corresponding author upon reasonable request.
